# Persistence Enhancement of a Promising Tick Repellent, Benzyl Isothiocyanate, by Yeast Microcarriers

**DOI:** 10.3390/molecules26226817

**Published:** 2021-11-11

**Authors:** Hui-Ju Kim, Ah-Hyeon Jeong, Ji-Hoon Lee, Jun-Hwan Park

**Affiliations:** Department of Bioenvironmental Chemistry, College of Agriculture and Life Sciences, Jeonbuk National University, Jeonju 54896, Korea; gmlwn0515@jbnu.ac.kr (H.-J.K.); dkgys123@jbnu.ac.kr (A.-H.J.)

**Keywords:** *Armoracia rusticana* root oil, *Haemaphysalis longicornis* nymphs, repellency, benzyl isothiocyanate, encapsulation, yeast cell

## Abstract

Phenethyl isothiocyanate isolated from *Armoracia rusticana* root oil and its derivatives were tested at different doses in a bioassay designed to evaluate repellency against individual *Haemaphysalis longicornis* nymphs. Among the tested compounds, benzyl isothiocyanate exhibited repellency against *H. longicornis* nymphs at the lowest dose of 0.00625 mg/cm^2^, followed by phenethyl isothiocyanate (0.0125 mg/cm^2^) and phenyl isothiocyanate (0.025 mg/cm^2^). The behavioral responses of *H. longicornis* nymphs exposed to benzyl isothiocyanate and phenethyl isothiocyanate indicated that the mode of action of these compounds can be mainly attributed to the vapor phase. Encapsulated benzyl isothiocyanate showed repellency up to 120 min post-application at 0.1 mg/cm^2^, whereas pure benzyl isothiocyanate showed repellency up to 60 min post-application at 0.1 mg/cm^2^. The present study suggests that benzyl isothiocyanate is a potential repellent for protection against *H. longicornis* nymphs, and encapsulation in yeast cells may enhance the repellency effect.

## 1. Introduction

The Asian longhorned tick, *Haemaphysalis longicornis* (Ixodida: Ixodidae), is an important livestock pest transmitting various tick-borne infectious diseases including severe fever with thrombocytopenia syndrome (SFTS). SFTS poses some of the most significant issues due to its high case fatality rate (6–30%) in humans [1]. SFTS is an emerging infectious disease caused by the SFTS virus (SFTSV), a member of the genus *Phlebovirus* [1]. As there are no specific medications or vaccines available for SFTS, control is mostly focused on targeting its primary vector, *H. longicornis*. *H. longicornis* is native to Eastern Asia (China, Japan, and South Korea) but has become common as an invasive tick in New Zealand and Australia [2]. Recently, *H. longicornis* was discovered in New Jersey and is now rapidly spreading throughout the United States of America [3]. The rapid spread of *H. longicornis* and *H. longicornis*-borne diseases, including SFTS, presents a potential worldwide threat.

The most effective way to prevent *H. longicornis*-borne diseases is to use tick repellents to avoid *H. longicornis* bites. Various natural products derived from botanical sources, including plant essential oils, have been researched and described as potential tick repellents [4]. Generally, the biological activity of essential oils depends on the content and composition of the major active compounds. Some volatile compounds from essential oils exhibit excellent repellent effects comparable to those of synthetic repellents, such as *N,N*-diethyl-meta-toluamide [5,6]. However, the inclusion of essential oils and volatile compounds in repellents remains limited owing to their poor stability, water solubility, and high volatility [7]. Therefore, strategies that address these problems are necessary if essential oils and volatile compounds are to be used commercially as repellents.

The use of encapsulation techniques to utilize essential oils or volatile compounds in commercial formulations is a promising method that can reduce their volatility and improve their efficiency [8]. The use of yeast cells (*Saccharomyces cerevisiae*) as the encapsulation vehicle is both cost-effective and simple [9]. This can usually be achieved by mixing water, yeast cells, and active substances under controlled temperatures without the need for specific additives [10]. The phospholipids in the yeast membrane act like a liposomal structure, which allows both hydrophobic and hydrophilic active ingredients to be encapsulated in the yeast cells [11]. In addition, baker’s yeast is a food-grade material and is non-toxic, so it can be used without negatively affecting human health [9].

The present research is focused on horseradish, *Armoracia rusticana*, which belongs to the Brassicaceae family. The root of *A. rusticana*, a pungent spice, is used worldwide as a flavoring agent [12]. These roots contain various isothiocyanates, which are known for their antifungal, antibacterial, anticancer, and insecticidal properties [13,14,15,16]. Although the biological activities of *A. rusticana* roots and isothiocyanates have been previously investigated, the potency of the root as a repellent against ixodid ticks has never been studied. Previous studies have shown that essential oil from *Gynandropsis gynandra* (Brassicales) exhibits tick-repellent effects [17]. The essential oil was found to contain 2.1% methyl isothiocyanate, but its contribution to ixodid tick-repellent activity has not been clarified. Furthermore, there have been no studies on the encapsulation of isothiocyanates in yeast cells. Therefore, the objectives of this study were to: (i) evaluate the repellent activity of *A. rusticana* root oil, (ii) isolate and identify the active compound from *A. rusticana* root oil, (iii) investigate the structure–activity relationships between the isolated compounds and their derivatives, and (iv) evaluate yeast cell-encapsulated benzyl isothiocyanate as a repellent in comparison with pure benzyl isothiocyanate.

## 2. Results and Discussion

### 2.1. Repellency of A. rusticana Root Oil

The repellency of *A. rusticana* root oil against *H. longicornis* nymphs was evaluated using a filter paper bioassay, the results of which are shown in Table 1. The median difference between *A. rusticana* root oil and the negative control was highly significant (*p* < 0.01) for doses greater than 0.025 mg/cm^2^. This indicates that repellent compounds exist in *A. rusticana* root oil. Therefore, the active component in *A. rusticana* root oil was isolated and identified.

### 2.2. Identification of the Active Component

The structure of the isolated compound was determined using spectroscopic analyses, including ^1^H-, ^13^C-, COSY-, DEPT-, and HMQC-NMR. The active compound was characterized as phenethyl isothiocyanate. MS (EI) *m*/*z* (%): 163 (44) [M^+^], 128 (0.7), 116 (0.2), 105 (19), 91 (100), 77 (9.2), 65 (9.8), 51 (6), 39 (4), 27 (1.3); C_9_H_9_NS. ^1^H-NMR (CDCl_3_): δ_H_ 2.985 (2H, *t*, *J* = 6.88 Hz), 3.707 (2H, *t*, *J* = 6.88 Hz), 7.203 (2H, *d*), 7.272 (2H, *t*), 7.336 (1H, *t*); ^13^C-NMR: δ 36.610 (C-3), 46.485 (C-2), 130.850 (C-1), 127.302 (C-7), 128.655 (C-5,9), 137.074 (C-4), 128.887 (C-6,8). The spectroscopic data for phenethyl isothiocyanate were largely consistent with previous data [18].

### 2.3. Repellency of Phenethyl Isothiocyanate Isolated from A. rusticana Root Oil and Its Derivatives

**Figure 1:**
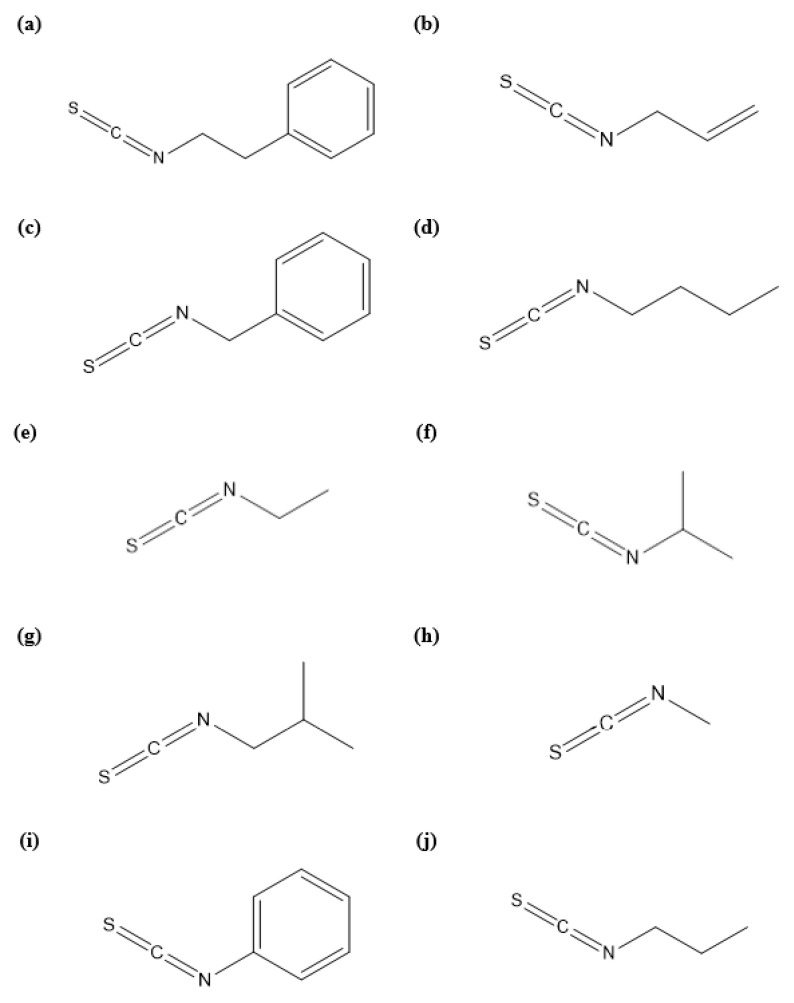
Chemical structures of phenethyl isothiocyanate (**a**) isolated from *Armoracia* *rusticana* root oil and its derivatives, allyl isothiocyanate (**b**), benzyl isothiocyanate (**c**), butyl isothiocyanate (**d**), ethyl isothiocyanate (**e**), isopropyl isothiocyanate (**f**), isobutyl isothiocyanate (**g**), methyl isothiocyanate (**h**), phenyl isothiocyanate (**i**), and propyl isothiocyanate (**j**).

To determine the relationships between phenethyl isothiocyanate derivatives and their repellent activities against *H. longicornis* nymphs, numerous derivatives were selected, including allyl isothiocyanate, benzyl isothiocyanate, butyl isothiocyanate, ethyl isothiocyanate, isobutyl isothiocyanate, isopropyl isothiocyanate, methyl isothiocyanate, phenyl isothiocyanate, and propyl isothiocyanate (Figure 1). Among these isothiocyanates, benzyl isothiocyanate exhibited repellent activity at the lowest dose (0.00625 mg/cm^2^), followed by phenethyl isothiocyanate (0.0125 mg/cm^2^) and phenyl isothiocyanate (0.025 mg/cm^2^) (Table 2). Allyl isothiocyanate, butyl isothiocyanate, ethyl isothiocyanate, isobutyl isothiocyanate, isopropyl isothiocyanate, methyl isothiocyanate, and propyl isothiocyanate did not show repellent activity even at the highest dose of 0.1 mg/cm^2^. Among the phenethyl isothiocyanate derivatives, the aromatic isothiocyanates showed repellent activity against *H. longicornis* nymphs, whereas aliphatic isothiocyanates did not. Similar results were obtained by Borek et al. [19], who observed that aromatic isothiocyanates (phenyl isothiocyanate, benzyl isothiocyanate, and 2-phenylethyl isothiocyanate) were more toxic to black vine weevil eggs than aliphatic isothiocyanates (methyl isothiocyanate, propyl isothiocyanate, and allyl isothiocyanate). In addition, Jang et al. [20] reported that aromatic isothiocyanates (2-phenylethyl, benzyl isothiocyanate, and 4-pentenyl isothiocyanate) showed higher potency than aliphatic isothiocyanates (3-butenyl isothiocyanate and 4-pentenyl isothiocyanate) against pathogenic bacteria. These results suggested that the presence of an aromatic ring in the isothiocyanate skeleton might play an important role in its repellent potency against *H. longicornis* nymphs. IR3535, which was used as a positive control in this study, showed repellency against *H. longicornis* nymphs at dosages up to 0.05 mg/cm^2^. Similar results were obtained by Wong et al. [21], who observed that synthetic IR3535 showed lower repellent activity against American dog ticks (Dermacentor variabilis) than did the active ingredients (myristicin, safrole, and terpinolene) in rosemary and nutmeg essential oils. Commercially available synthetic repellents may not be satisfactorily effective against ticks because many of them have been developed to protect humans from mosquitos [22,23]. In our study, benzyl isothiocyanate, phenyl isothiocyanate, and phenethyl isothiocyanate exhibited repellent activity at lower doses than IR3535, indicating that these substances are potentially useful for the control of *H. longicornis* nymphs.

**Figure 2:**
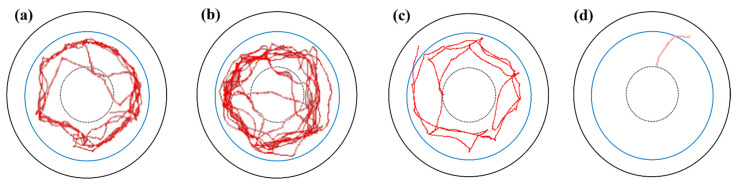
Representative tracks showing the behavior of *Haemaphysalis longicornis* nymphs on filter paper treated with phenethyl isothiocyanate (**a**), benzyl isothiocyanate (**b**), IR3535 (**c**), or negative control (ethanol) (**d**) on the outer circle (i.e., outside the blue line).

Most bioassays for tick repellency are unable to effectively discriminate between the repellency caused by olfaction (non-contact repellency) and that caused by tactile chemoreception (contact repellency) [24]. In principle, in the filter paper bioassay used here, the complex sensory apparatus of *H. longicornis* nymphs could come into contact with the treated zone of the experimental arena; however, according to our observations using video-tracking software, *H. longicornis* nymphs came within approximately 2–4 mm of the phenethyl isothiocyanate, benzyl isothiocyanate, or IR3535-treated zone but did not touch it (Figure 2). Similar results were obtained in an experiment on *Ixodes ricinus* that used a Y-tube olfactometer bioassay with DEET-treated filter paper. These findings indicated that olfaction was involved in the observed repellency of benzyl isothiocyanate and phenethyl isothiocyanate.

### 2.4. Confirmation of Encapsulated Benzyl Isothiocyanate

**Figure 3:**
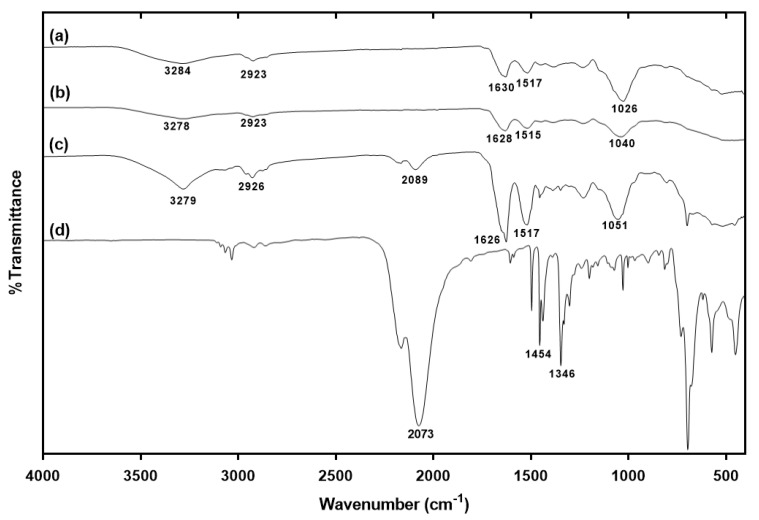
Fourier transform infrared spectroscopy (FT−IR) spectra of non−plasmolyzed yeast cells (**a**), plasmolyzed yeast cells (**b**), benzyl isothiocyanate−loaded plasmolyzed yeast cells (**c**), and pure benzyl isothiocyanate (**d**).

**Figure 4:**
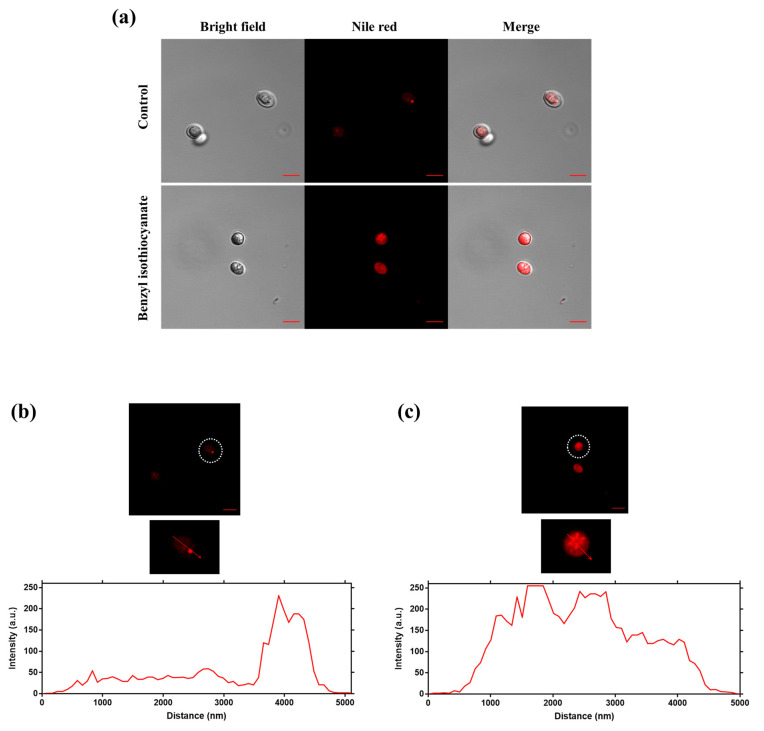
Confocal micrographs and fluorescence intensity profiles of benzyl isothiocyanate-loaded plasmolyzed yeast cells. The top row of (**a**) represents the bright field, Nile Red, and merge images of the control (plasmolyzed yeast cell). The bottom row of (**a**) represents the bright field, Nile Red, and merge images of benzyl isothiocyanate-loaded plasmolyzed yeast cells. Line-scan profiles of fluorescence intensity for the control (plasmolyzed yeast cell) and benzyl isothiocyanate-loaded plasmolyzed yeast cells are shown in (**b**,**c**), respectively.

The loading capacity of the benzyl isothiocyanate-loaded plasmolyzed yeast cells was 58.1%. The FT-IR spectra of both the non-plasmolyzed and plasmolyzed yeast cells showed a prominent adsorption band at 3700–3000 cm^−1^ (OH vibration of yeast polysaccharides) (Figure 3). The absorption bands of the non-plasmolyzed yeast cells at 1630, 1517, and 1026 cm^−1^ could be attributed to the bands of amide I, amide II, and mannans, respectively. In the plasmolyzed yeast cells, the absorption bands of amide I, amide II, and mannan were shifted and their intensity decreased. These changes resulted from the degradation of the yeast protein and the transition of the degraded protein to the unfolded state [10,25]. The FT-IR results obtained for the non-plasmolyzed and plasmolyzed yeast cells were in accordance with the results of previous studies [10,25,26]. The FT-IR spectrum of benzyl isothiocyanate showed prominent adsorption bands at 2089 cm^–1^ (N=C=S stretching vibration) and 1345–1600 cm^−1^ (benzene ring). All the observed adsorption bands correlate well with those in the literature [27]. Absorption bands appeared at 2073 and 1345–1600 cm^−1^ in the spectra of the benzyl isothiocyanate-loaded plasmolyzed yeast cells. These bands originated from the N=C=S group and the benzene ring of benzyl isothiocyanate. These results indicated the presence of benzyl isothiocyanate in the plasmolyzed yeast cells. The confocal micrographs and fluorescence intensity profiles of the plasmolyzed yeast cells and the benzyl isothiocyanate-loaded yeast cells stained with Nile Red clearly showed that both cell types remained intact and that they successfully encapsulated benzyl isothiocyanate (Figure 4). The plasmolyzed yeast cells had a slightly red color due to the presence of lipids in the cell membrane. After the benzyl isothiocyanate was loaded, the color of the cells turned deep red, confirming that the benzyl isothiocyanate entered and remained in the plasmolyzed yeast cells.

### 2.5. Repellency of Encapsulated Benzyl Isothiocyanate

Encapsulated benzyl isothiocyanate showed repellency up to 120 min post-application at 0.1 mg/cm^2^, whereas pure benzyl isothiocyanate showed repellency up to 60 min post-application at 0.1 mg/cm^2^ (Table 3). The decrease in the repellency of the essential oil and its main constituents over time may be because of their high volatility. Pavela [28] showed that the prolonged repellency of *Carum carvi* and *Thymus vulgaris* essential oils against *Meligethes aeneus* might be because of the presence of less volatile compounds, such as thymol and carvone. Renkema et al. [29] also showed that the prolonged repellent effect of peppermint oil against *Drosophila suzukii* could be related to its consistently low release rate. Benzyl isothiocyanate is an aromatic compound with high volatility [30]. Thus, this characteristic can explain the significant decrease in the repellent efficacy of benzyl isothiocyanate over time. Yeast cell walls, which are mainly composed of β-1,3-glucan, chitin, and a mannoprotein layer, can function as barriers to preserve a substance and control its release [10,31]. Thus, yeast cell walls support the prolonged repellent effects of encapsulated benzyl isothiocyanate by protecting the active substance from rapid volatilization. The plasmolysis of yeast cells using NaCl can provide more intracellular space for active substances by expelling water and water-soluble components, including amino acids, enzymes, nucleic acids, and proteins, from the cell [31]. In addition, using plasmolysis as a pretreatment before the encapsulation process could improve the oxidative stability of the active substance [11,32]. This is explained by the fact that, as the intracellular space of yeast cells increases owing to plasmolysis pretreatment, the active substance can be located in the cells rather than on the cell surfaces, which can lead to improved oxidative stability [32].

The main limitation of this study is that it did not consider the stability of benzyl isothiocyanate-loaded yeast cells. However, there is reliable evidence to suggest that the encapsulation of plant-derived materials within yeast cells can provide oxidative and thermal stability. Thus, further studies on the stability of benzyl isothiocyanate-loaded yeast cells are necessary for exploring the potential of this technique.

## 3. Conclusions

Phenethyl isothiocyanate isolated from *A. rusticana* root oil and its derivatives showed potential repellent effects against *H. longicornis* nymphs, particularly benzyl isothiocyanate. The ability of yeast cell-encapsulated benzyl isothiocyanate to repel *H. longicornis* nymphs was also confirmed. Encapsulation with plasmolyzed yeast cells prolonged the strong repellency effect of benzyl isothiocyanate by 180 m. These results suggest that yeast cell-encapsulated benzyl isothiocyanate is a promising repellent for the management of *H. longicornis* nymphs. Further studies on the stability of benzyl isothiocyanate encapsulated in yeast cells are now necessary.

## 4. Methods

### 4.1. Chemicals

Allyl isothiocyanate (95%), benzyl isothiocyanate (98%), butyl isothiocyanate (98%), ethyl isothiocyanate (97%), isobutyl isothiocyanate (97%), isopropyl isothiocyanate (98%), methyl isothiocyanate (97%), Nile Red, phenyl isothiocyanate (99%), propyl isothiocyanate (98%), and 3-[*N*-butyl-*N*-acetyl] aminopropionic acid ethyl ester (IR3535) (95%) were purchased from Tokyo Chemical Industry Co. (Tokyo, Japan) or Sigma-Aldrich Co. (St. Louis, MO, USA). Yeast cells were purchased from Biozoa Biological Supply (Seoul, Korea).

### 4.2. Plant Essential Oils

*A. rusticana* roots cultivated in Australia were purchased from a local market in Seoul, South Korea, in January 2020. Essential oil of *A. rusticana* roots were prepared by steam distillation for 6 h in a Clevenger-type apparatus [33]. The obtained essential oil was dehydrated using anhydrous magnesium sulfate, and then it was evaporated to dryness for 15 min using a rotary evaporator (EYELA, Tokyo, Japan) at 30 °C.

### 4.3. Ticks

*H. longicornis* nypmhs were collected from Seogwipo-si, Jejudo, South Korea by dragging a white cotton cloth (100 × 90 cm) over the grass at ground level. Ticks were identified as *H. longicornis* using a taxonomic key [34]. All collected nymphs were maintained at 25 °C and 70% relative humidity (RH) in an incubator.

### 4.4. Isolation and Identification of Active Compounds

*A. rusticana* root oil (20 g) was loaded into a silica gel column and gradually eluted with a mixed organic solvent, hexane:ethyl acetate (8:2, *v*/*v*) to obtain 15 fractions. The obtained fractions were concentrated using a rotary evaporator (N-3 NM, EYELA, Tokyo, Japan) and examined using thin-layer chromatography to obtain nine fractions (AR1–9). The repellent effects of the nine fractions against *H. longicornis* nymphs were evaluated using a filter paper bioassay with a concentration of 0.1 mg/cm^2^. AR5 fraction exhibited potent activity against *H. longicornis* nymphs. The active AR5 fraction was further chromatographed using preparative high-performance liquid chromatography (LC–908, JAI Co., Ltd., Tokyo, Japan) using a GS series column with hexane/chloroform (1/3, *v*/*v*) at a flow rate of 3 mL/min. Finally, the repellent compound AR52 (1.31 g) was isolated. The chemical structure of the isolated compound was determined using electron ionization mass spectroscopy (EI-MS), one-dimensional nuclear magnetic resonance (NMR) spectra (^1^H-NMR, ^13^C-NMR, and DEPT-NMR), and two-dimensional NMR spectra (^1^H-^1^H COSY-NMR [JNM-ECA600, JEOL, Japan]; ^1^H-NMR at 600 MHz and ^13^C-NMR at 150 MHz; nuclear observations: ^1^H, ^13^C, ^15^N, ^29^Si, ^31^P, etc.; and solvent CDCl_3_).

### 4.5. Repellent Activity Bioassays

#### Filter Paper Bioassay

**Figure 5:**
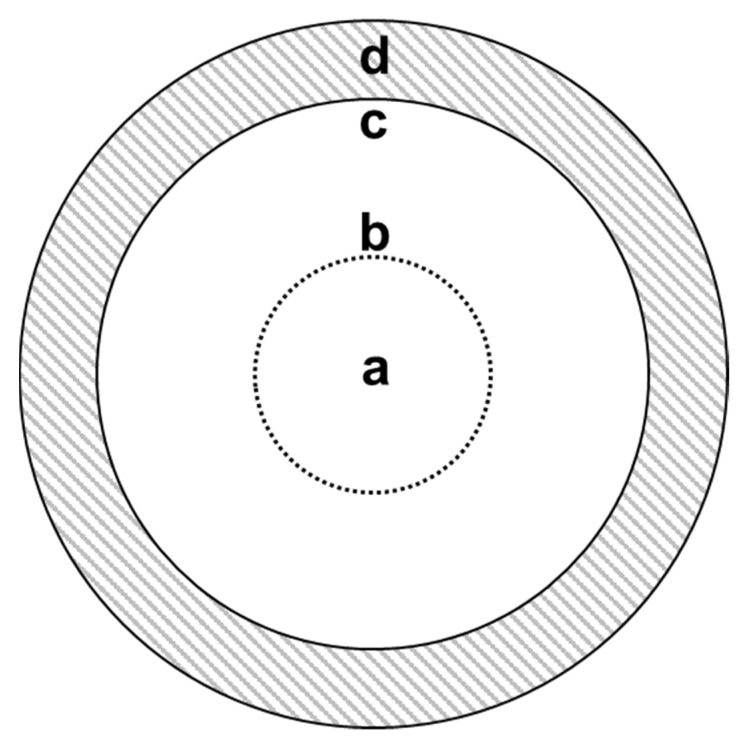
Filter paper arena used in the repellent bioassays. (**a**) Drop zone with a single *Haemaphysalis longicornis* nymph, (**b**) start line, (**c**) finish line, (**d**) treated circle.

The repellent effects of *A. rusticana* root oil and the isolated compound and its derivatives against *H. longicornis* nymphs were tested using a filter paper bioassay as described by Del Fabbro and Nazzi [35] and Wong et al. [21] with slight modifications. Two concentric circles were drawn on the test filter paper, one with a radius of 1.5 cm (start line) and the other with a radius of 3.5 cm (finish line). Various concentrations of each sample (0.1, 0.05, 0.025, 0.0125, 0.00625, and 0.003125 mg/cm^2^) were prepared by dissolving in ethanol, and each sample was applied on a 1.0 cm-wide donut-shaped filter paper. In each case, after evaporating the solvent (ethanol) for 20 min, the donut-shaped filter paper was attached to the border of finish line B on the test filter paper to prevent each sample from spreading to the non-treated area (Figure 5). Ethanol was used as the negative control. Each *H. longicornis* nymph was placed in the center of the arena with a soft paintbrush (5 mm wide). Each *H. longicornis* nymph was tested first on the control filter paper and subsequently on the test filter paper. Ticks were observed until they crossed the finish line, and the time spent between the start and finish lines was recorded. If the test sample was found to be repellent at 0.05 mg/cm^2^, it was also tested at 0.025 mg/cm^2^; if it was also repellent at 0.0125 and 0.00625 mg/cm^2^, it was tested at 0.003125 mg/cm^2^. IR3535 was used as a positive control for comparison with individual compounds in their repellency against *H. longicornis* nymphs [36]. If a tick did not cross the start line before 150 s, it was eliminated from the experiment. If a tick took longer than 500 s to approach the finish line, the time was recorded as 500 s. For the filter paper bioassay of encapsulated benzyl isothiocyanate, the sample was applied on a 1.0 cm-wide donut-shaped filter paper at 0.1 mg/cm^2^ and then dried for 60, 120, or 180 h. If the test sample was found to be repellent at 60 min post-treatment, it was also tested at 120 min post-treatment; if it was also repellent at 120 min post-treatment, it was tested at 180 min post-treatment. These experiments were performed at 25 °C and 70% RH. A total of 30 ticks were used in each treatment and control group. During the experiment, the behavior of each nymph was recorded using a webcam (c270, Logitech), and the video data were analyzed using the video-tracking software Ethowatcher^®^ [37].

### 4.6. Encapsulation in Yeast Cells

The plasmolysis of yeast cells for encapsulation was conducted as described by Kavetsou et al. [9] and Shi et al. [11]. Briefly, the cells were washed with distilled water and mixed with 5% (*w*/*v*) NaCl solution at a ratio of 1:2 (*w*/*w*). The suspension was stirred at 200 rpm at 60 °C for 48 h. Then, the yeast cells were harvested using centrifugation at 8000 rpm for 10 min, washed with distilled water until NaCl and unwanted substances were removed, and freeze-dried for 48 h. The plasmolyzed yeast cells were mixed with benzyl isothiocyanate and distilled water with a ratio of 1:1.5:6 (*w*/*w*) and stirred at 160 rpm at 40 °C for 48 h. Then, the mixture was centrifuged at 8000 rpm for 10 min. After removing the supernatants, the remaining microcapsules were washed six times with distilled water and freeze-dried for 48 h.

### 4.7. Analysis of Encapsulated Benzyl Isothiocyanate

#### Loading Capacity (LC)

**Figure 6:**
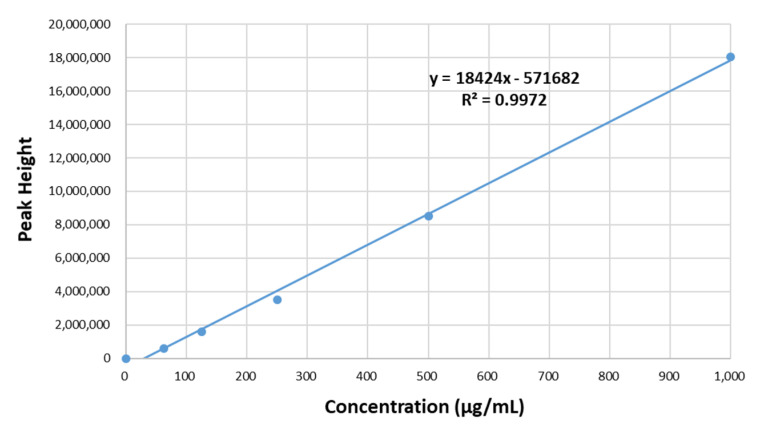
GC-MS calibration curve for standard benzyl isothiocyanate.

Quantification of benzyl isothiocyanate in loaded microcapsules was performed by gas chromatography–mass spectrometry (GC-MS) (GCMS-QP2010 Ultra, Shimadzu, Kyoto, Japan) using a DB-5 (0.25 mm film) fused silica capillary column (30 m × 0.25 mm i.d. × 0.25 μm thickness) [38]. For the GC-MS, the carrier gas was helium at a flow rate of 0.8 mL/min, the column temperature was 60–200 °C rising at a rate of 2.0 °C/min, and the temperature of the ion source was 210 °C. Mass spectra were taken at 70, and a mass analyzer was used for a scan area of 20–400 atomic mass units. Standard benzyl isothiocyanate solutions in the range of 62.5–1000 μg/mL were created in order to prepare a calibration curve of peak area versus benzyl isothiocyanate concentration (Figure 6). Benzyl isothiocyanate was extracted from 100 mg microcapsules using 10 mL of methanol. Then, the extracts were bath sonicated for 15 min and centrifuged at 8000 rpm for 10 min. The supernatant was measured by GC-MS, and the peak area was recorded. The amount of benzyl isothiocyanate was calculated using the calibration curve. LC (%) was calculated as follows [9]:

(1) $$\mathrm{LC}\;\left(\%\right)=\frac{\mathrm{entrapped}\;\mathrm{benzyl}\;\mathrm{isothiocyanate}\;\mathrm{weight}}{\mathrm{loaded}\;\mathrm{microcapsule}\;\mathrm{weight}}\times 100$$

### 4.8. Fourier Transform Infrared Spectroscopy (FT-IR)

The infrared spectra of yeast cells (non-plasmolyzed and plasmolyzed), benzyl isothiocyanate-loaded plasmolyzed yeast cells, and pure benzyl isothiocyanate were recorded at wavenumbers of 400–4000 cm^−1^ using attenuated total reflectance (ATR)-FT-IR (PerkinElmer, Frontier, Waltham, MA, USA) [10,25,26].

### 4.9. Confocal Imaging

The microstructures of yeast cells, plasmolyzed yeast cells, and plasmolyzed yeast cells encapsulated by benzyl isothiocyanate were observed using a confocal laser scanning microscope (LSM 510 META, Carl Zeiss, Jena, Germany) with a 100× oil immersion objective as described by Fu et al. [39] with slight modifications. Before analysis, 1 mg of benzyl isothiocyanate microcapsules or plasmolyzed yeast cells dissolved in 1 mL distilled water was mixed with Nile Red solution (10 μL) to stain the benzyl isothiocyanate and lipids. The excitation and emission wavelengths of Nile Red are 514 nm and 609 nm, respectively.

### 4.10. Statistical Analysis

As the filter paper bioassay results had a non-normal distribution, the median was used as a central tendency measure and the nonparametric Mann–Whitney U-test was used for hypothesis testing (SPSS version 12.0, New York, NY, UUSA).

## Figures and Tables

**Table 1:** Repellency of *Armoracia rusticana* root oil against *Haemaphysalis longicornis* nymphs at different doses using a filter paper bioassay ^a^.

Dose (mg/cm^2^)	Treatment Median (s) ^b^	Negative Control Median (s) ^b^	*p*-Value ^c^	Treatment/Negative Control ^d^
0.1	227.5	12.5	< 0.01	18.2
0.05	221.0	13.0	< 0.01	17.0
0.025	219.0	15.0	< 0.01	14.6
0.0125	34.50	14.5	n.s. ^e^	2.4

^a^ A total of 30 ticks were used in each treatment. ^b^ The median time spent by ticks between the start line and finish line. ^c^ Statistical significance of the difference between the medians of the treatment and negative control (nonparametric Mann–Whitney U-test). ^d^ Ratio between the medians of the treatment and negative control. ^e^ No statistically significant difference.

**Table 2:** Repellent effects of phenethyl isothiocyanate isolated from *Armoracia rusticana* root oil and its derivatives against *Haemaphysalis longicornis* nymphs at different doses using a filter paper bioassay ^a^.

Samples	Dose (mg/cm^2^)	Treatment Median (s) ^b^	Negative Control Median (s) ^b^	*p*-Value ^c^	Treatment/Negative Control ^d^
Allyl isothiocyanate	0.1	15.5	14.5	n.s. ^e^	1.2
Benzyl isothiocyanate	0.1	500.0	11.0	<0.01	45.5
	0.05	500.0	12.5	<0.01	40.0
	0.025	500.0	12.0	<0.01	41.7
	0.0125	244.0	13.5	<0.01	18.1
	0.00625	76.5	14.0	<0.01	5.5
	0.003125	24.0	16.5	n.s.	1.5
Butyl isothiocyanate	0.1	15.5	14.5	n.s.	1.1
Ethyl isothiocyanate	0.1	16.5	12.5	n.s.	1.3
Isobutyl isothiocyanate	0.1	16.5	14.0	n.s.	1.2
Isopropyl isothiocyanate	0.1	14.5	13.0	n.s.	1.1
Methyl isothiocyanate	0.1	19.0	14.0	n.s.	1.4
Phenyl isothiocyanate	0.1	139.5	13.0	<0.01	10.7
	0.05	48.0	14.0	<0.05	3.4
	0.025	39.5	14.0	<0.05	2.8
	0.0125	8.5	13.0	n.s.	0.7
Phenethyl isothiocyanate	0.1	500.0	12.0	<0.01	41.7
	0.05	500.0	12.5	<0.01	40.0
	0.025	500.0	14.0	<0.01	35.7
	0.0125	202.5	13.5	<0.01	15.0
	0.00625	26.5	12.0	n.s.	2.2
Propyl isothiocyanate	0.1	18.0	12.5	n.s.	1.4
Positive control(IR3535)	0.1	292.5	15.0	<0.01	19.5
	0.05	145.5	14.5	<0.01	10.0
	0.025	17.5	14.0	n.s.	1.3

^a^ A total of 30 ticks were used in each treatment. ^b^ The median time spent by ticks between the start line and finish line. ^c^ Statistical significance of the difference between the medians of the treatment and negative control (nonparametric Mann–Whitney U-test). ^d^ Treatment median/negative control median, ^e^ No statistically significant difference.

**Table 3:** Repellent effects of pure and encapsulated benzyl isothiocyanate against *Haemaphysalis longicornis* nymphs using a filter paper bioassay ^a^.

Samples ^b^	Time Post-Treatment (min)	Treatment Median (s) ^c^	Negative Control Median (s) ^c^	*p*-Value ^d^	Treatment /Negative Control ^e^
Pure benzyl isothiocyanate	60	39.5	13.5	<0.01	2.9
	120	11.0	10.5	n.s. ^f^	1.1
Encapsulated benzyl isothiocyanate	60	49.0	13.0	<0.01	3.8
	120	27.0	14.0	<0.01	1.9
	180	12.5	11.0	n.s.	1.1

^a^ A total of 30 ticks were used in each treatment. ^b^ Dose of 0.1 mg/cm^2^. ^c^ The median time spent by ticks between the start line and finish line. ^d^ Statistical significance of the difference between the medians of the treatment and negative control (nonparametric Mann–Whitney U-test). ^e^ Treatment median/negative control median. ^f^ No statistically significant difference.

## Data Availability

The data that support the findings of this study are present in the main manuscript or supplementary information. Additional data related to this paper may be obtained from the corresponding author on reasonable request.

## Sample Availability

Samples of the compounds are available from the authors.

## References

[1] Luo L.M., Zhao L., Wen H.L., Zhang Z.T., Liu J.W., Fang L.Z., Xue Z.F., MA D.Q., Zhang X.S., Ding S.J. (2015). *Haemaphysalis longicornis* ticks as reservoir and vector of severe fever with thrombocytopenia syndrome virus in China. Emerg. Infect. Dis..

[2] Heath A.C.G. (2016). Biology, ecology and distribution of the tick, *Haemaphysalis longicornis* Neumann (Acari: Ixodidae) in New Zealand. N. Z. Vet. J..

[3] Wormser G.P., McKenna D., Piedmonte N., Vinci V., Egizi A.M., Backenson B., Falco R.C. (2020). First recognized human bite in the United States by the Asian longhorned tick, *Haemaphysalis longicornis*. Clin. Infect. Dis..

[4] Benelli G., Pavela R. (2018). Repellence of essential oils and selected compounds against ticks—A systematic review. Acta Trop..

[5] Bissinger B.W., Roe R.M. (2010). Tick repellents: Past, present, and future. Pestic. Biochem. Physiol..

[6] Jordan R.A., Schulze T.L., Dolan M.C. (2012). Efficacy of plant-derived and synthetic compounds on clothing as repellents against *Ixodes scapularis* and *Amblyomma americanum* (Acari: Ixodidae). J. Med. Entomol..

[7] Turek C., Stintzing F.C. (2013). Stability of essential oils: A review. Compr. Rev. Food Sci. Food Saf..

[8] Majeed H., Bian Y.Y., Ali B., Jamil A., Majeed U., Khan Q.F., Iqbal K.J., Shoemaker C.F., Fang Z. (2015). Essential oil encapsulations: Uses, procedures, and trends. Rsc Adv..

[9] Kavetsou E. (2019). Encapsulation of *Mentha pulegium* essential oil in yeast cell microcarriers: An approach to environmentally friendly pesticides. J. Agric. Food Chem..

[10] Paramera E.I., Konteles S.J., Karathanos V.T. (2011). Stability and release properties of curcumin encapsulated in *Saccharomyces cerevisiae*, β-cyclodextrin and modified starch. Food Chem..

[11] Shi G., Rao L., Yu H., Xiang H., Yang H., Ji R. (2008). Stabilization and encapsulation of photosensitive resveratrol within yeast cell. Int. J. Pharm..

[12] Agneta R., Möllers C., Rivelli A.R. (2013). Horseradish (*Armoracia rusticana*), a neglected medical and condiment species with a relevant glucosinolate profile: A review. Genet. Resour. Crop. Evol..

[13] Wu X., Zhou Q.H., Xu K. (2009). Are isothiocyanates potential anti-cancer drugs. Acta Pharmacol. Sin..

[14] Dufour V., Stahl M., Baysse C. (2015). The antibacterial properties of isothiocyanates. Microbiology.

[15] Kara M., Soylu E.M. (2020). Assessment of glucosinolate-derived isothiocyanates as potential natural antifungal compounds against citrus sour rot disease agent *Geotrichum citri-aurantii*. J. Phytopathol..

[16] Du Y., Grodowitz M.J., Chen J. (2020). Insecticidal and enzyme inhibitory activities of isothiocyanates against red imported fire ants, *Solenopsis invicta*. Biomolecules.

[17] Lwande W., Ndakala A.J., Hassanali A., Moreka L., Nyandat E., Ndungu M., Amiani H., Gitu P.M., Malonza M.M., Punyua D.K. (1999). Gynandropsis gynandra essential oil and its constituents as tick (*Rhipicephalus appendiculatus*) repellents. Phytochemistry.

[18] Chen H., Wang C., Ye J., Zhou H., Chen X. (2012). Antimicrobial activities of phenethyl isothiocyanate isolated from horseradish. Nat. Prod. Res..

[19] Borek V., Elberson L.R., McCaffrey J.P., Morra M.J. (1998). Toxicity of isothiocyanates produced by glucosinolates in Brassicaceae species to black vine weevil eggs. J. Agric. Food Chem..

[20] Jang M., Hong E., Kim G.H. (2010). Evaluation of antibacterial activity of 3-butenyl, 4-pentenyl, 2-phenylethyl, and benzyl isothiocyanate in Brassica vegetables. J. Food Sci..

[21] Wong C., Crystal K., Coats J. (2021). Three molecules found in rosemary or nutmeg essential oils repel ticks (Dermacentor variabilis) more effectively than DEET in a no-human assay. Pest Manag. Sci..

[22] Schreck C.E., Fish D., McGovern T.P. (1995). Activity of repellents applied to skin for protection against *Amblyomma americanum* and *Ixodes scapularis* ticks (Acari: Ixodidae) *J*. Am. Mosq. Control Assoc.-Mosq. News.

[23] Adenubi O.T., McGaw L.J., Eloff J.N., Naidoo V. (2018). *In vitro* bioassays used in evaluating plant extracts for tick repellent and acaricidal properties: A critical review. Vet. Parasitol..

[24] Carroll J.F., Klun J.A., Debboun M. (2005). Repellency of deet and SS220 applied to skin involves olfactory sensing by two species of ticks. Med. Vet. Entomol..

[25] Burattini E., Cavagna M., Dell’Anna R., Campeggi F.M., Monti F., Rossi F., Torriani S. (2008). A FTIR microspectroscopy study of autolysis in cells of the wine yeast *Saccharomyces cerevisiae*. Vib. Spectrosc..

[26] Karaman K. (2020). Characterization of *Saccharomyces cerevisiae* based microcarriers for encapsulation of black cumin seed oil: Stability of thymoquinone and bioactive properties. Food Chem..

[27] Li W., Liu X., Yang Q., Zhang N., Du Y., Zhu H. (2015). Preparation and characterization of inclusion complex of benzyl isothiocyanate extracted from papaya seed with β-cyclodextrin. Food Chem..

[28] Pavela R. (2011). Insecticidal and repellent activity of selected essential oils against of the pollen beetle, *Meligethes aeneus* (Fabricius) adults. Ind. Crops Prod..

[29] Renkema J.M., Wright D., Buitenhuis R., Hallett R.H. (2016). Plant essential oils and potassium metabisulfite as repellents for *Drosophila suzukii* (Diptera: Drosophilidae). Sci. Rep..

[30] Uppal S., Kumar R., Sareen S., Kaur K., Mehta S.K. (2020). Biofabrication of cerium oxide nanoparticles using emulsification for an efficient delivery of Benzyl isothiocyanate. Appl. Surf. Sci..

[31] Normand V., Dardelle G., Bouquerand P.E., Nicolas L., Johnston D.J. (2005). Flavor encapsulation in yeasts: Limonene used as a model system for characterization of the release mechanism. J. Agric. Food Chem..

[32] Kavosi M., Mohammadi A., Shojaee Aliabadi S., Khaksar R., Hosseini S.M. (2018). Characterization and oxidative stability of purslane seed oil microencapsulated in yeast cells biocapsules. J. Sci. Food Agric..

[33] Park J.H., Lee H.S. (2018). Acaricidal target and mite indicator as color alteration using 3,7-dimethyl-2,6-octadienal and its derivatives derived from *Melissa officinalis* leaves. Sci. Rep..

[34] Barker S.C., Walker A.R. (2014). Ticks of Australia. The species that infest domestic animals and humans. Zootaxa.

[35] Del Fabbro S., Nazzi F. (2013). From chemistry to behavior. Molecular structure and bioactivity of repellents against *Ixodes ricinus* ticks. PLoS ONE.

[36] Tavares M., da Silva M.R.M., de Siqueira L.B.D.O., Rodrigues R.A.S., Bodjolle-d’Almeida L., Dos Santos E.P., Ricci-Júnior E. (2018). Trends in insect repellent formulations: A review. Int. J. Pharm..

[37] Junior C.F.C., Pederiva C.N., Bose R.C., Garcia V.A., Lino-de-Oliveira C., Marino-Neto J. (2012). ETHOWATCHER: Validation of a tool for behavioral and video-tracking analysis in laboratory animals. Comput. Biol. Med..

[38] Abdel-Kader M.S., Khamis E.H., Foudah A.I., Alqarni M.H. (2018). GC quantitative analysis of benzyl isothiocyanate in *Salvadora persica* roots extract and dental care herbal products. Saudi Pharm. J..

[39] Fu J., Guan J., Sun C., Zhou D., Zhu B. (2021). Encapsulation of Antarctic krill oil in yeast cell microcarriers: Evaluation of oxidative stability and in vitro release. Food Chem..

